# AusTraits, a curated plant trait database for the Australian flora

**DOI:** 10.1038/s41597-021-01006-6

**Published:** 2021-09-30

**Authors:** Daniel Falster, Rachael Gallagher, Elizabeth H. Wenk, Ian J. Wright, Dony Indiarto, Samuel C. Andrew, Caitlan Baxter, James Lawson, Stuart Allen, Anne Fuchs, Anna Monro, Fonti Kar, Mark A. Adams, Collin W. Ahrens, Matthew Alfonzetti, Tara Angevin, Deborah M. G. Apgaua, Stefan Arndt, Owen K. Atkin, Joe Atkinson, Tony Auld, Andrew Baker, Maria von Balthazar, Anthony Bean, Chris J. Blackman, Keith Bloomfield, David M. J. S. Bowman, Jason Bragg, Timothy J. Brodribb, Genevieve Buckton, Geoff Burrows, Elizabeth Caldwell, James Camac, Raymond Carpenter, Jane A. Catford, Gregory R. Cawthray, Lucas A. Cernusak, Gregory Chandler, Alex R. Chapman, David Cheal, Alexander W. Cheesman, Si-Chong Chen, Brendan Choat, Brook Clinton, Peta L. Clode, Helen Coleman, William K. Cornwell, Meredith Cosgrove, Michael Crisp, Erika Cross, Kristine Y. Crous, Saul Cunningham, Timothy Curran, Ellen Curtis, Matthew I. Daws, Jane L. DeGabriel, Matthew D. Denton, Ning Dong, Pengzhen Du, Honglang Duan, David H. Duncan, Richard P. Duncan, Marco Duretto, John M. Dwyer, Cheryl Edwards, Manuel Esperon-Rodriguez, John R. Evans, Susan E. Everingham, Claire Farrell, Jennifer Firn, Carlos Roberto Fonseca, Ben J. French, Doug Frood, Jennifer L. Funk, Sonya R. Geange, Oula Ghannoum, Sean M. Gleason, Carl R. Gosper, Emma Gray, Philip K. Groom, Saskia Grootemaat, Caroline Gross, Greg Guerin, Lydia Guja, Amy K. Hahs, Matthew Tom Harrison, Patrick E. Hayes, Martin Henery, Dieter Hochuli, Jocelyn Howell, Guomin Huang, Lesley Hughes, John Huisman, Jugoslav Ilic, Ashika Jagdish, Daniel Jin, Gregory Jordan, Enrique Jurado, John Kanowski, Sabine Kasel, Jürgen Kellermann, Belinda Kenny, Michele Kohout, Robert M. Kooyman, Martyna M. Kotowska, Hao Ran Lai, Etienne Laliberté, Hans Lambers, Byron B. Lamont, Robert Lanfear, Frank van Langevelde, Daniel C. Laughlin, Bree-Anne Laugier-Kitchener, Susan Laurance, Caroline E. R. Lehmann, Andrea Leigh, Michelle R. Leishman, Tanja Lenz, Brendan Lepschi, James D. Lewis, Felix Lim, Udayangani Liu, Janice Lord, Christopher H. Lusk, Cate Macinnis-Ng, Hannah McPherson, Susana Magallón, Anthony Manea, Andrea López-Martinez, Margaret Mayfield, James K. McCarthy, Trevor Meers, Marlien van der Merwe, Daniel J. Metcalfe, Per Milberg, Karel Mokany, Angela T. Moles, Ben D. Moore, Nicholas Moore, John W. Morgan, William Morris, Annette Muir, Samantha Munroe, Áine Nicholson, Dean Nicolle, Adrienne B. Nicotra, Ülo Niinemets, Tom North, Andrew O’Reilly-Nugent, Odhran S. O’Sullivan, Brad Oberle, Yusuke Onoda, Mark K. J. Ooi, Colin P. Osborne, Grazyna Paczkowska, Burak Pekin, Caio Guilherme Pereira, Catherine Pickering, Melinda Pickup, Laura J. Pollock, Pieter Poot, Jeff R. Powell, Sally A. Power, Iain Colin Prentice, Lynda Prior, Suzanne M. Prober, Jennifer Read, Victoria Reynolds, Anna E. Richards, Ben Richardson, Michael L. Roderick, Julieta A. Rosell, Maurizio Rossetto, Barbara Rye, Paul D. Rymer, Michael A. Sams, Gordon Sanson, Hervé Sauquet, Susanne Schmidt, Jürg Schönenberger, Ernst-Detlef Schulze, Kerrie Sendall, Steve Sinclair, Benjamin Smith, Renee Smith, Fiona Soper, Ben Sparrow, Rachel J. Standish, Timothy L. Staples, Ruby Stephens, Christopher Szota, Guy Taseski, Elizabeth Tasker, Freya Thomas, David T. Tissue, Mark G. Tjoelker, David Yue Phin Tng, Félix de Tombeur, Kyle Tomlinson, Neil C. Turner, Erik J. Veneklaas, Susanna Venn, Peter Vesk, Carolyn Vlasveld, Maria S. Vorontsova, Charles A. Warren, Nigel Warwick, Lasantha K. Weerasinghe, Jessie Wells, Mark Westoby, Matthew White, Nicholas S. G. Williams, Jarrah Wills, Peter G. Wilson, Colin Yates, Amy E. Zanne, Graham Zemunik, Kasia Ziemińska

**Affiliations:** 1 Evolution & Ecology Research Centre, School of Biological, Earth, and Environmental Sciences, UNSW Sydney, Sydney, Australia; 2 Department of Biological Sciences, Macquarie University, Sydney, Australia; 3 CSIRO Land and Water, Canberra, Australia; 4 NSW Department of Primary Industries, Orange, Australia; 5 Centre for Australian National Biodiversity Research (a joint venture between Parks Australia and CSIRO), Canberra, ACT Australia; 6 Swinburne University of Technology, Hawthorn, Australia; 7 Hawkesbury Institute for the Environment, Western Sydney University, Sydney, Australia; 8 La Trobe University, Bundoora, Australia; 9 Centre for Rainforest Studies, School for Field Studies, Yungaburra, Queensland 4872 Australia; 10 University of Melbourne, Melbourne, Australia; 11 The Australian National University, Canberra, Australia; 12 NSW Department of Planning Industry and Environment, Parramatta, Australia; 13 Southern Cross University, Lismore, Australia; 14 Department of Botany and Biodiversity Research, University of Vienna, Vienna, Austria; 15 Queensland Herbarium, Toowong, Australia; 16 University of Tasmania, Hobart, Australia; 17 Imperial College, London, United Kingdom; 18 Research Centre for Ecosystem Resilience, Australian Institute of Botanical Science, Royal Botanic Gardens and Domain Trust, Sydney, Australia; 19 James Cook University, Douglas, Australia; 20 Charles Sturt University, Bathurst, Australia; 21 School of Biological Sciences, Monash University, Clayton, Australia; 22 Centre of Excellence for Biosecurity Risk Analysis, The University of Melbourne, Melbourne, Australia; 23 University of Adelaide, Adelaide, Australia; 24 King’s College London, London, United Kingdom; 25 University of Western Australia, Crawley, Australia; 26 College of Science and Engineering, James Cook University, Cairns, QLD Australia; 27 Department of Agriculture, Sydney, Australia; 28 Western Australian Herbarium, Keiran McNamara Conservation Science Centre, Department of Biodiversity, Conservation and Attractions, Western Australia, Kensington, Australia; 29 Centre for Environmental Management, School of Health & Life Sciences, Federation University, Mount Helen, Australia; 30 Royal Botanic Gardens, Richmond, Kew United Kingdom; 31 Fenner School of Environment and Society, The Australian National University, Canberra, Australia; 32 Lincoln University, Lincoln, New Zealand; 33 University of Technology Sydney, Sydney, Australia; 34 Environment Department, Alcoa of Australia, Huntly, Western Australia Australia; 35 School of Marine and Tropical Biology, James Cook University, Douglas, Australia; 36 School of Agriculture, Food and Wine, University of Adelaide, Adelaide, Australia; 37 Lanzhou University, Lanzhou, China; 38 Institute for Forest Resources & Environment of Guizhou, Guizhou University, Guiyang, China; 39 Institute for Applied Ecology, University of Canberra, ACT, 2617 Canberra, Australia; 40 National Herbarium of New South Wales, Australian Institute of Botanical Science, Royal Botanic Gardens and Domain Trust, Sydney, Australia; 41 School of Biological Sciences, The University of Queensland, St Lucia, Australia; 42 Melbourne Water, Melbourne, Australia; 43 Queensland University of Technology, Brisbane, Australia; 44 Departamento de Ecologia, Universidade Federal do Rio Grande do Norte, Natal, Natal – RN, Brazil; 45 Pathways Bushland and Environment Consultancy, Sydney, Australia; 46 Department of Plant Sciences, University of California, Davis, USA; 47 USDA-ARS, WMSRU, Fort Collins, Colorado, 80526 USA; 48 Biodiversity and Conservation Science, Department of Biodiversity, Conservation and Attractions, Kensington, WA Australia; 49 Curtin University, Bentley, Australia; 50 University of New England, Armidale, Australia; 51 Terrestrial Ecosystem Research Network, The School of Biological Sciences, The University of Adelaide, Adelaide, SA 5005 Australia; 52 School of Ecosystem and Forest Sciences, The University of Melbourne, Melbourne, Australia; 53 Tasmanian Institute of Agriculture, University of Tasmania, Hobart, Australia; 54 arks Australia, Department of Agriculture, Water and the Environment, Hobart, Australia; 55 School of Life and Environmental Sciences, The University of Sydney, Camperdown, Australia; 56 Berowa NSW, Berowa, Australia; 57 Nanchang Institute of Technology, Nanchang, China; 58 Western Australian Herbarium, Biodiversity and Conservation Science, Department of Biodiversity, Conservation and Attractions, Kensington, Western Australia Australia; 59 Universidad Autonoma de Nuevo Leon, San Nicolás de los Garza, Mexico; 60 Australian Wildlife Conservancy, Sydney, Australia; 61 State Herbarium of South Australia, Botanic Gardens and State Herbarium, Hackney Road, Adelaide, SA 5000 Australia; 62 NSW Rural Fire Service, Sydney, Australia; 63 Department of Environment, Land, Water and Planning, Victoria, Australia; 64 Department of Plant Ecology and Ecosystems Research, University of Goettingen, Göttingen, Germany; 65 University of Canterbury, Christchurch, New Zealand; 66 Institut de recherche en biologie végétale, Université de Montréal, 4101 Sherbrooke Est, Montréal, H1X 2B2 Canada; 67 Ecology and Evolution, Research School of Biology, Australian National University, Canberra, Australia; 68 Wildlife Ecology & Conservation Group, Wageningen University, Wageningen, The Netherlands; 69 Department of Botany, University of Wyoming, Laramie, WY 82071 USA; 70 Royal Botanic Garden Edinburgh, Edinburgh, United Kingdom; 71 Fordham University, New York City, NY USA; 72 AMAP (Botanique et Modélisation de l’Architecture des Plantes et des Végétations), Université de Montpellier, CIRAD, CNRS, INRA, IRD, Montpellier, France; 73 University of Otago, Dunedin, New Zealand; 74 Environmental Research Institute, University of Waikato, Hamilton, New Zealand; 75 University of Auckland, Auckland, New Zealand; 76 Laboratorio Nacional de Ciencias de la Sostenibilidad, Instituto de Ecología, Universidad Nacional Autónoma de México, Ciudad de México, Mexico; 77 Manaaki Whenua – Landcare Research, Lincoln, 7640 New Zealand; 78 Cumberland Ecology, Cumberland, Australia; 79 Linkoping University, Linkoping, Sweden; 80 Currency Creek Arboretum, Currency Creek, Australia; 81 Estonian University of Life Sciences, Tartu, Estonia; 82 Leistershire County Council, Leicester, United Kingdom; 83 Division of Natural Sciences, New College of Florida, Sarasota, USA; 84 Graduate School of Agriculture, Kyoto University, Kyoto, Japan; 85 Centre for Ecosystem Science, School of Biological, Earth, and Environmental Sciences, UNSW, Sydney, Australia; 86 University of Sheffield, Department of Animal and Plant Sciences, Sheffield, United Kingdom; 87 Istanbul Technical University, Eurasia Institute of Earth Sciences, Istanbul, Turkey; 88 Department of Civil and Environmental Engineering, Massachusetts Institute of Technology, Cambridge, USA; 89 School of Environment and Science, Griffith University, Brisbane, Australia; 90 Greening Australia, Brisbane, Australia; 91 Department of Biology, McGill University, Montréal, Canada; 92 Western Australian Herbarium, Department of Biodiversity, Conservation and Attractions, Western Australia, Kensington, Australia; 93 School of Agriculture and Food Science, University of Queensland, St Lucia, Australia; 94 Max-Planck Institute for Biogeochemistry, Jena, Germany; 95 Rider University, Lawrence Township, Lawrenceville, NJ USA; 96 McGill University, Montreal, Canada; 97 Environmental and Conservation Sciences, Murdoch University, Murdoch, Australia; 98 TERRA Teaching and Research Centre, Gembloux Agro-Bio Tech, University of Liege, Gembloux, Belgium; 99 Xishuangbanna Tropical Botanic Garden, Yunnan, China; 100 Centre for Integrative Ecology, School of Life and Environmental Sciences, Deakin University, Burwood, Australia; 101 Faculty of Agriculture, University of Peradeniya, Peradeniya, 20400 Sri Lanka; 102 National Herbarium of NSW and Royal Botanic Gardens and Domain Trust, Sydney, Australia; 103 Department of Biological Sciences, George Washington University, Washington, DC 20052 USA; 104 Present Address: Hawkesbury Institute for the Environment, Western Sydney University, Sydney, Australia; 105 Present Address: Department of Biology, University of Miami, Coral Gables, Florida 33146 USA, George Washington University, Washington, DC 20052 USA

**Keywords:** Ecology, Evolution, Ecology

## Abstract

We introduce the AusTraits database - a compilation of values of plant traits for taxa in the Australian flora (hereafter AusTraits). AusTraits synthesises data on 448 traits across 28,640 taxa from field campaigns, published literature, taxonomic monographs, and individual taxon descriptions. Traits vary in scope from physiological measures of performance (e.g. photosynthetic gas exchange, water-use efficiency) to morphological attributes (e.g. leaf area, seed mass, plant height) which link to aspects of ecological variation. AusTraits contains curated and harmonised individual- and species-level measurements coupled to, where available, contextual information on site properties and experimental conditions. This article provides information on version 3.0.2 of AusTraits which contains data for 997,808 trait-by-taxon combinations. We envision AusTraits as an ongoing collaborative initiative for easily archiving and sharing trait data, which also provides a template for other national or regional initiatives globally to fill persistent gaps in trait knowledge.

## Abstract (summary)

**Table:** 

Measurement(s)	plant trait
Technology Type(s)	digital curation
Sample Characteristic - Organism	Viridiplantae
Sample Characteristic - Location	Australia

Machine-accessible metadata file describing the reported data: 10.6084/m9.figshare.14545755

## Background & Summary

Species traits are essential for comparing ecological strategies among plants, both within any given vegetation and across environmental space or evolutionary lineages^1–4^. Broadly, a trait is any measurable property of a plant capturing aspects of its structure or function^5–8^. Traits thereby provide useful indicators of species’ behaviours in communities and ecosystems, regardless of their taxonomy^8–10^. Through global initiatives the volume of available trait information for plants has grown rapidly in the last two decades^11,12^. However, the geographic coverage of trait measurements across the globe is patchy, limiting detailed analyses of trait variation and diversity in some regions, and, more generally, development of theory accounting for the diversity of plant strategies.

One such region where trait data is sparsely documented is Australia; a continent with a flora of c. 28,900 native vascular plant taxa^13^ (including species, subspecies, varietas and forma). While significant investment has been made in curating and digitising herbarium collections and observation records in Australia over the last two decades (e.g. The Australian Virtual Herbarium houses ~7 million specimen occurrence records; https://avh.ala.org.au), no complementary resource yet exists for consolidating information on plant traits. Moreover, relatively few Australian species are represented in the leading global databases. For example, the international TRY database^12^ has measurements for only 3830 Australian species across all collated traits. This level of species coverage limits our ability to use traits to understand and ultimately manage Australian vegetation^14^. While initiatives such as TRY^12^ and the Open Traits Network^15^ are working towards global synthesis of trait data, a stronger representation of Australian plant taxa in these efforts is essential, especially given the high richness and endemicity of this continental flora, and the unique contribution this makes to global floral diversity^16,17^.

**Fig. 1:**
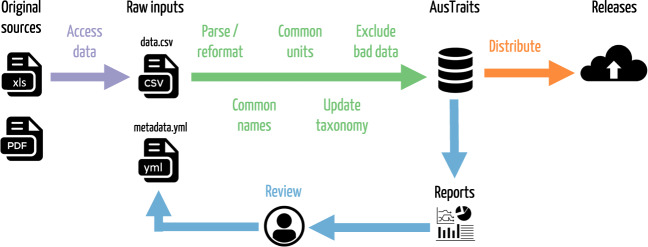
The data curation pathway used to assemble the AusTraits database. Trait measurements are accessed from original data sources, including published floras and field campaigns. Features such as variable names, units and taxonomy are harmonised to a common standard. Versioned releases are distributed to users, allowing the dataset to be used and re-used in a reproducible way.

Here we introduce the AusTraits database (hereafter AusTraits), a compilation of plant traits for the Australian flora. Currently, AusTraits draws together 283 distinct sources and contains 997,808 measurements spread across 448 different traits for 28,640 taxa. To assemble AusTraits from diverse primary sources and make data available for reuse, we needed to overcome three main types of challenges (Fig. 1): (1) Accessing data from diverse original sources, including field studies, online databases, scientific articles, and published taxonomic floras; (2) Harmonising these diverse sources into a federated resource, with common taxon names, units, trait names, and data formats; and (3) Distributing versions of the data under suitable license. To meet this challenge, we developed a workflow which draws on emerging community standards and our collective experience building trait databases.

By providing a harmonised and curated dataset on 448 plant traits, AusTraits contributes substantially to filling the gap in Australian and global biodiversity resources. Prior to the development of AusTraits, data on Australian plant traits existed largely as a series of disconnected datasets collected by individual laboratories or initiatives.

AusTraits has been developed as a standalone database, rather than as part of the existing global database TRY^12^, for three reasons. First, we sought to establish an engaged and localised community, actively collaborating to enhance coverage of plant trait data within Australia. We envisioned that a community would form more readily to fill gaps in national knowledge of traits with local ownership of the resource. While we will never have a counterfactual, a vibrant community excited to be part of this initiative has indeed been established and coverage is much higher for Australian species than has been achieved since TRY’s inception. Local ownership also aligns well with funding opportunities and national research priorities, and enables database coordinators to progress at their own speed. Second, we wanted to apply an entirely open-source approach to the aggregation workflow. All the code and raw files used to create the compiled database are available, and this database is freely available via a third party data repository (Zenodo) which is itself built for long term data archiving, with an established API. Finally, we targeted primary data sources, where possible, whereas TRY accepts aggregated datasets. The hope was that this would increase data quality, by removing intermediaries and easier identification of duplicates.

While independent, the overall structure of AusTraits is similar to that of TRY, ensuring the two databases will be interoperable. Both databases are founded on similar principles and terminology^18,19^. Increasingly, researchers and biodiversity portals are seeking to connect diverse datasets^15^, which is possible if they share a common foundation.

We envision AusTraits as an on-going collaborative initiative for easily archiving and sharing trait data about the Australian flora. Open access to a comprehensive resource like this will generate significant new knowledge about the Australian flora across multiple scales of interest, as well as reduce duplication of effort in the compilation of plant trait data, particularly for research students and government agencies seeking to access information on traits. In coming years, AusTraits will continue to be expanded, with integrations into other biodiversity platforms and expansion of coverage into historically neglected plant lineages in trait science, such as pteridophytes (lycophytes and ferns). Further, through international initiatives, such as the Open Traits Network, linkages are being forged between plant datasets and a variety of other organismal databases^15^.

## Methods

### Primary sources

AusTraits version 3.0.2 was assembled from 283 distinct sources, including published papers, field measurements, glasshouse and field experiments, botanical collections, and taxonomic treatments. Initially we identified a list of candidate traits of interest, then identified primary sources containing measurements for these traits, before contacting authors for access. As the compilation grew, we expanded the list of traits considered to include any measurable quantity that had been quantified for at least a moderate number of taxa (n > 20).

For a small subset of sources from herbaria, providing a text description of taxa, we used regular expressions in R to extract measurements of traits from the text. A variety of expressions were developed to extract height, leaf/seed dimensions and growth form. Error checking was completed on approximately 60% of mined measurements by visually inspecting the extracted values relative to the textual descriptions.

### Trait definitions

A full list of traits and their sources appears in Supplementary Table 1^20–354^ . The list of sources in AusTraits was developed gradually as new datasets were incorporated, drawing from original source publications and a published thesaurus of plant characteristics^19^. We categorised traits based on the tissue where it is measured (bark, leaf, reproductive, root, stem, whole plant) and the type of measurement (allocation, life history, morphology, nutrient, physiological). Version 3.0.2 of AusTraits includes 358 numeric and 90 categorical traits.

### Database structure

The schema of AusTraits broadly follows the principles of the established Observation and Measurement Ontology^18^ in that, where available, trait data are connected to contextual information about the collection (e.g. location coordinates, light levels, whether data were collected in the field or lab) and information about the methods used to derive measurements (e.g. number of replicates, equipment used). The database contains 11 elements, as described in Table 1. This format was developed to include information about the trait measurements, taxon, methods, sites, contextual information, people involved, and citation sources.

**Table 1:** Main elements of the harmonised AusTraits database. See Tables 2–8 for details on each component.

Element	Contents
traits	A table containing measurements of plant traits.
sites	A table containing observations of site characteristics associated with information in ‘traits’. Cross referencing between the two dataframes is possible using combinations of the variables ‘dataset_id’, ‘site_name’.
contexts	A table containing observations of contextual characteristics associated with information in ‘traits’. Cross referencing between the two dataframes is possible using combinations of the variables ‘dataset_id’, ‘context_name’.
methods	A table containing details on methods with which data were collected, including time frame and source.
excluded_data	A table of data that did not pass quality test and so were excluded from the master dataset.
taxa	A table containing details on taxa associated with information in ‘traits’. This information has been sourced from the APC (Australian Plant Census) and APNI (Australian Plant Name Index) and is released under a CC-BY3 license.
definitions	A copy of the definitions for all tables and terms. Information included here was used to process data and generate any documentation for the study.
sources	Bibtex entries for all primary and secondary sources in the compilation.
contributors	A table of people contributing to each study.
taxonomic_updates	A table of all taxonomic changes implemented in the construction of AusTraits. Changes are determined by comparing against the APC (Australian Plant Census) and APNI (Australian Plant Name Index).
build_info	A description of the computing environment used to create this version of the dataset, including version number, git commit and R session_info.

For storage efficiency, the main table of traits contains relatively little information (Table 2), but can be cross linked against other tables (Tables 3–8) using identifiers for dataset, site, context, observation, and taxon (Table 1). The dataset_id is ordinarily the surname of the first author and year of publication associated with the source’s primary citation (e.g. Blackman_2014). Trait values were also recorded as being one of several possible value types (value_type) (Table 9), reflecting the type of measurement submitted by the contributor, as different sources provide different levels of detail. Possible values include raw_value, individual_mean, site_mean, multisite_mean, expert_mean, experiment_mean. Further details on the methods used for collecting each trait are provided in a methods table (Table 5).

**Table 2:** Structure of the traits table, containing measurements of plant traits.

key	value
dataset_id	Primary identifier for each study contributed into AusTraits; most often these are scientific papers, books, or online resources. By default should be name of first author and year of publication, e.g. ‘Falster_2005’.
taxon_name	Currently accepted name of taxon in the Australian Plant Census or in the Australian Plant Name Index.
site_name	Name of site where individual was sampled. Cross-references to identical columns in ‘sites’ and ‘traits’.
context_name	Name of contextual senario where individual was sampled. Cross-references to identical columns in ‘contexts’ and ‘traits’.
observation_id	A unique identifier for the observation, useful for joining traits coming from the same ‘observation_id’. These are assigned automatically, based on the ‘dataset_id’ and row number of the raw data.
trait_name	Name of trait sampled.
value	Measured value.
unit	Units of the sampled trait value after aligning with AusTraits standards.
date	Date sample was taken, in the format ‘yyyy-mm-dd’, but with days and months only when specified.
value_type	A categorical variable describing the type of trait value recorded.
replicates	Number of replicate measurements that comprise the data points for the trait for each measurement. A numeric value (or range) is ideal and appropriate if the value type is a ‘mean’, ‘median’, ‘min’ or ‘max’. For these value types, if replication is unknown the entry should be ‘unknown’. If the value type is ‘raw_value’ the replicate value should be 1. If the value type is ‘expert_mean’, ‘expert_min’, or ‘expert_max’ the replicate value should be ‘na’.
original_name	Name given to taxon in the original data supplied by the authors

**Table 3:** Structure of the sites table, containing observations of site characteristics associated with information in traits.

key	value
dataset_id	Primary identifier for each study contributed into AusTraits; most often these are scientific papers, books, or online resources. By default should be name of first author and year of publication, e.g. ‘Falster_2005’.
site_name	Name of site where individual was sampled. Cross-references to identical columns in ‘sites’ and ‘traits’.
site_property	The site characteristic being recorded. Name should include units of measurement, e.g. ‘longitude (deg)’. Ideally we have at least these variables for each site - ‘longitude (deg)’, ‘latitude (deg)’, ‘description’.
value	Measured value.

**Table 4:** Structure of the contexts table, containing observations of contextual characteristics associated with information in traits.

key	value
dataset_id	Primary identifier for each study contributed into AusTraits; most often these are scientific papers, books, or online resources. By default should be name of first author and year of publication, e.g. ‘Falster_2005’.
context_name	Name of contextual senario where individual was sampled. Cross-references to identical columns in ‘contexts’ and ‘traits’.
context_property	The contextual characteristic being recorded. Name should include units of measurement, e.g. ‘CO2 concentration (ppm)’.
value	Measured value.

**Table 5:** Structure of the methods table, containing details on methods with which data were collected, including time frame and source.

key	value
dataset_id	Primary identifier for each study contributed into AusTraits; most often these are scientific papers, books, or online resources. By default should be name of first author and year of publication, e.g. ‘Falster_2005’.
trait_name	Name of trait sampled. Allowable values specified in the table ‘traits’.
methods	A textual description of the methods used to collect the trait data. Whenever available, methods are taken near-verbatim from referenced source. Methods can include descriptions such as ‘measured on botanical collections’, ‘data from the literature’, or a detailed description of the field or lab methods used to collect the data.
year_collected_start	The year data collection commenced.
year_collected_end	The year data collection was completed.
description	A 1–2 sentence description of the purpose of the study.
collection_type	A field to indicate where the majority of plants on which traits were measured were collected - in the ‘field’, ‘lab’, ‘glasshouse’, ‘botanical collection’, or ‘literature’. The latter should only be used when the data were sourced from the literature and the collection type is unknown.
sample_age_class	A field to indicate if the study was completed on ‘adult’ or ‘juvenile’ plants.
sampling_strategy	A written description of how study sites were selected and how study individuals were selected. When available, this information is copied verbatim from a published manuscript. For botanical collections, this field ideally indicates which records were ‘sampled’ to measure a specific trait.
source_primary_citation	Citation for primary source. This detail is generated from the primary source in the metadata.
source_primary_key	Citation key for primary source in ‘sources’. The key is typically of format ‘Surname_year’.
source_secondary_citation	Citations for secondary source. This detail is generated from the secondary source in the metadata.
source_secondary_key	Citation key for secondary source in ‘sources’. The key is typically of format ‘Surname_year’.

**Table 6:** Structure of the taxonomic_updates table, of all taxonomic changes implemented in the construction of AusTraits. Changes are determined by comparing against the APC (Australian Plant Census) and APNI (Australian Plant Name Index).

key	value
dataset_id	Primary identifier for each study contributed into AusTraits; most often these are scientific papers, books, or online resources. By default should be name of first author and year of publication, e.g. ‘Falster_2005’.
original_name	Name given to taxon in the original data supplied by the authors
cleaned_name	Name of the taxon after implementing any changes encoded for this taxon in the metadata file for the correpsonding ‘dataset_id’.
taxonIDClean	Where it could be identified, the ‘taxonID’ of the ‘cleaned_name’ for this taxon in the APC.
taxonomicStatusClean	Taxonomic status of the taxon identified by ‘taxonIDClean’ in the APC.
alternativeTaxonomicStatusClean	The status of alternative records with the name ‘cleaned_name’ in the APC.
acceptedNameUsageID	ID of the accepted name for taxon in the APC or APNI.
taxon_name	Currently accepted name of taxon in the APC or in the APNI .

**Table 7:** Structure of the taxa table, containing details on taxa associated with information in the traits table. This information has been sourced from the APC (Australian Plant Census) and APNI (Australian Plant Name Index) and is released under a CC-BY3 license.

key	value
taxon_name	Currently accepted name of taxon in the APC or in the APNI .
source	Source of taxnonomic information, either APC or APNI.
acceptedNameUsageID	ID of the accepted name for taxon in the APC or APNI.
scientificNameAuthorship	Authority for taxon indicated under taxon_name.
taxonRank	Rank of the taxon.
taxonomicStatus	Taxonomic status of the taxon.
family	Family of the taxon.
genus	Genus of the taxon.
taxonDistribution	Known distribution of the taxon, by state.
ccAttributionIRI	Source of taxonomic information.

**Table 8:** Structure of the contributors table, of people contributing to each study.

key	value
dataset_id	Primary identifier for each study contributed into AusTraits; most often these are scientific papers, books, or online resources. By default should be name of first author and year of publication, e.g. ‘Falster_2005’.
name	Name of contributor
institution	Last known institution or affiliation
role	Their role in the study

**Table 9:** Possible value types of trait records.

key	value
raw_value	Value is a direct measurement
site_min	Value is the minimum of measurements on multiple individuals of the taxon at a single site
site_mean	Value is the mean or median of measurements on multiple individuals of the taxon at a single site
site_max	Value is the maximum of measurements on multiple individuals of the taxon at a single site
multisite_min	Value is the minimum of measurements on multiple individuals of the taxon across multiple sites
multisite_mean	Value is the mean or median of measurements on multiple individuals of the taxon across multiple sites
multisite_max	Value is the maximum of measurements on multiple individuals of the taxon across multiple sites
expert_min	Value is the minimum observed for a taxon across its range or in this particular dataset, as estimated by an expert based on their knowledge of the taxon. Data fitting this category include estimates from floras that represent a taxon’s entire range.
expert_mean	Value is the mean observed for a taxon across its range or in this particular dataset, as estimated by an expert based on their knowledge of the taxon. Data fitting this category include estimates from floras that represent a taxon’s entire range, and values for categorical variables obtained from a reference book, or identified by an expert.
expert_max	Value is the maximum observed for a taxon across its range or in this particular dataset, as estimated by an expert based on their knowledge of the taxon. Data fitting this category include estimates from floras that represent a taxon’s entire range.
experiment_min	Value is the minimum of measurements from an experimental study either in the field or a glasshouse
experiment_mean	Value is the mean or median of measurements from an experimental study either in the field or a glasshouse
experiment_max	Value is the maximum of measurements from an experimental study either in the field or a glasshouse
individual_mean	Value is a mean of replicate measurements on an individual (usually for experimental ecophysiology studies)
individual_max	Value is a maximum of replicate measurements on an individual (usually for experimental ecophysiology studies)
literature_source	Value is a site or multi-site mean that has been sourced from an unknown literature source
unknown	Value type is not currently known

### Harmonisation

To harmonise each source into the common AusTraits format we applied a reproducible and transparent workflow (Fig. 1), written in R^355^, using custom code, and the packages tidyverse^356^, yaml^357^, remake^358^, knitr^359^, and rmarkdown^360^. In this workflow, we performed a series of operations, including reformatting data into a standardised format, generating observation ids for each set of linked measurements, transforming variable names into common terms, transforming data into common units, standardising terms (trait values) for categorical variables, encoding suitable metadata, and flagging data that did not pass quality checks. Details from each primary source were saved with minimal modification into two plain text files. The first file, data.csv, contains the actual trait data in comma-separated values format. The second file, metadata.yml, contains relevant metadata for the study, as well as options for mapping trait names and units onto standard types, and any substitutions applied to the data in processing. These two files provide all the information needed to compile each study into a standardised AusTraits format. Successive versions of AusTraits iterate through the steps in Fig. 1, to incorporate new data and correct identified errors, leading to a high-quality, harmonised dataset.

After importing a study, we generated a detailed report which summarised the study’s metadata and compared the study’s data values to those collected by other studies for the same traits. Data for continuous and categorical variables are presented in scatter plots and tables respectively. These reports allow first the AusTraits data curator, followed by the data contributor, to rapidly scan the metadata to confirm it has been entered correctly and the trait data to ensure it has been assigned the correct units and their categorical traits values are properly aligned with AusTraits trait values.

### Taxonomy

We developed a custom workflow to clean and standardise taxonomic names using the latest and most comprehensive taxonomic resources for the Australian flora: the Australian Plant Census (APC)^13^ and the Australian Plant Name Index (APNI)^361^. These resources document all known taxonomic names for Australian plants, including currently accepted names and synonyms. While several automated tools exist for updating taxonomy, such as taxize^362^, these do not currently include up to date information for Australian taxa. Updates were completed in two steps. In the first step, we used both direct and then fuzzy matching (with up to 2 characters difference) to search for an alignment between reported names and those in three name sets: 1) All accepted taxa in the APC, 2) All known names in the APC, 3) All names in the APNI. Names were aligned without name authorities, as we found this information was rarely reported in the raw datasets provided to us. Second, we used the aligned name to update any outdated names to their current accepted name, using the information provided in the APC. If a name was recorded as being both an accepted name and an alternative (e.g. synonym) we preferred the accepted name, but also noted the alternative records. For phrase names, when a suitable match could not be found, we manually reviewed near matches via web portals such as the Atlas of Living Australia to find a suitable match. The final resource reports both the original and the updated taxon name alongside each trait record (Table 2), as well as an additional table summarising all taxonomic name changes (Table 6) and further information from the APC and APNI on all taxa included (Table 7). Any changes in taxonomy are exposed within the compiled dataset, enabling researchers to review these as needed.

## Data Records

### Access

Static versions of AusTraits, including version 3.0.2 used in this descriptor, are available via Zenodo^363^. Data is released under a CC-BY license enabling reuse with attribution – being a citation of this descriptor and, where possible, original sources. Deposition within Zenodo helps makes the dataset consistent with FAIR principles^364^. As an evolving data product, successive versions of AusTraits are being released, containing updates and corrections. Versions are labeled using semantic versioning to indicate the change between versions^365^. As validation (see Technical Validation, below) and data entry are ongoing, users are recommended to pull data from release, to ensure results in their downstream analyses remain consistent as the database is updated.

The R package austraits (https://github.com/traitecoevo/austraits) provides easy access to data and examples on manipulating data (e.g. joining tables, subsetting) for those using this platform.

### Data coverage

**Fig. 2:**
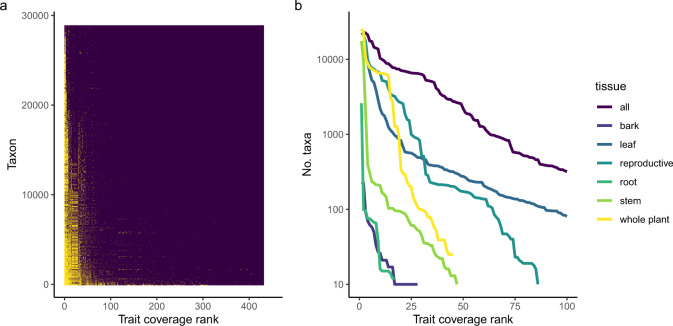
Coverage of traits by taxa. (**a**) Matrix showing the coverage of taxa for each trait, with yellow indicating presence of data. The figure was generated with a subset of 500 randomly selected taxa. (**b**) Number of taxa with data for first 100 traits for all traits and separated by tissue.

The number of accepted vascular plant taxa in the APC (as of May 2020) is around 28,981^13^. Version 3.0.2 of AusTraits includes at least one record for 26,852 taxa (~93% of known taxa). Five traits (leaf_length, leaf_width, plant_height, life_history, plant_growth_form) have records for more than 50% of known species (Fig. 2a). Across all traits, the median number of taxa with records is 62. Supplementary Table 1 shows the number of studies, taxa, and families with data in AusTraits, as well as the number of geo-referenced records, for each trait. Looking across traits and tissue categories, coverage declined gradually, with moderate coverage(>20%) for more than 50 traits (Fig. 2). Coverage for root, stem and bark traits declined much faster than trait measurements for other plant tissues (Fig. 2b).

**Fig. 3:**
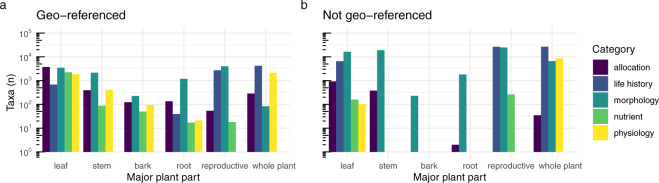
Number of taxa with trait records by plant tissue and trait category, for data that are (**a**) Geo-referenced, and (**b**) Not geo-referenced. Many records without a geo-reference come from botanical collections, such as floras.

The most common traits are non geo-referenced records from floras; these are trait values representing a continental or region mean (or spread) and hence are not linked to a location. Yet, geo-referenced records were available for several traits for more than 10% of the flora (Fig. 3a). Coverage is notably higher for geo-referenced measurements of some tissues and trait types - such as bark stems and roots - relative to non-geo-referenced measurements (Fig. 3).

**Fig. 4:**
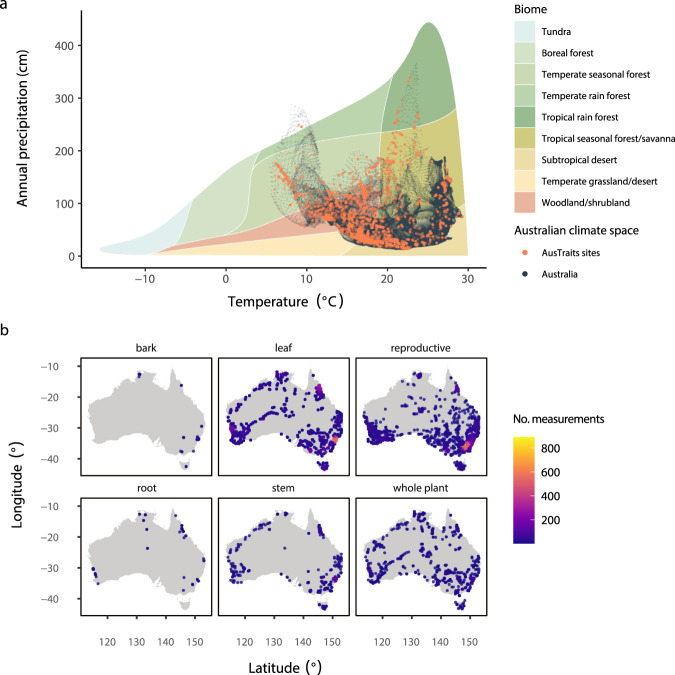
Coverage of geo-referenced trait records across Australian climatic and geographic space for traits in different categories. (**a**) AusTraits’ sites (orange) within Australia’s precipitation-temperature space (dark-grey) superimposed upon Whittaker’s classification of major biomes by climate^370^. Climate data were extracted at 10" resolution from WorldClim^371^. (**b**) Locations of geo-referenced records for different plant tissues.

Trait records are spread across the climate space of Australia (Fig. 4a), as well as geographic locations (Fig. 4b). As with most data in Australia, the density of records was somewhat concentrated around cities or roads in remote regions.

**Fig. 5:**
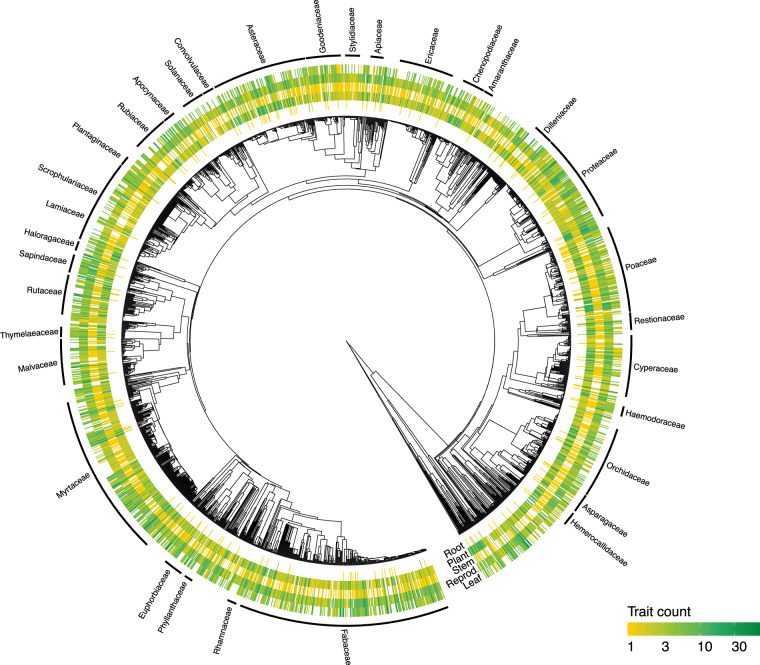
Phylogenetic distribution of trait data in AusTraits for a subset of 2000 randomly sampled taxa. The heatmap colour intensity denotes the number of traits measured within a family for each plant tissue. The most widespread family names (with more than ten taxa) are labelled on the edge of the tree.

Overall trait coverage across an estimated phylogenetic tree of Australian plant species is relatively unbiased (Fig. 5), though there are some notable exceptions. One exception is for root traits, where taxa within Poaceae have large amounts of information available relative to other plant families. A cluster of taxa within the family Myrtaceae which are largely from Western Australia have little leaf information available.

**Fig. 6:**
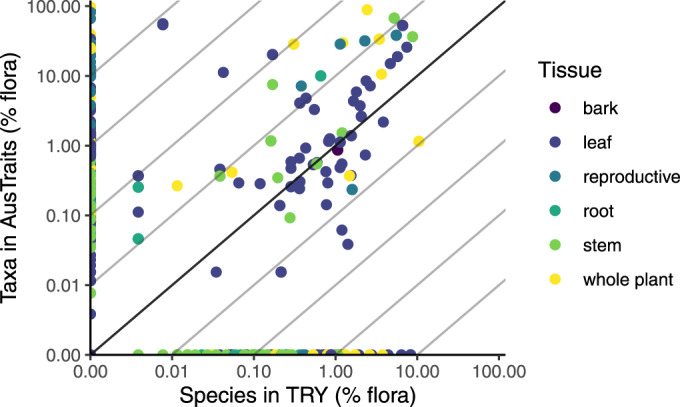
The number of taxa with trait records in AusTraits and global TRY database (accessed 28 May 2020). Each point shows a separate trait.

Comparing coverage in AusTraits to the global database TRY, there were 76 traits overlapping. Of these, AusTraits tended to contain records for more taxa, but not always; multiple traits had more than 10 times the number of taxa represented in AusTraits (Fig. 6). However, there were more records in TRY for 25 traits, in particular physiological leaf traits. Many traits were not overlapping between the two databases (Fig. 6). We noted that AusTraits includes more seed and fruit nutrient data; possibly reflecting the interest in Australia in understanding how fruit and seeds are provisioned in nutrient-depauperate environments. AusTraits includes more categorical values, especially variables documenting different components of species’ fire response strategies, reflecting the importance of fire in shaping Australian communities and the research to document different strategies species have evolved to succeed in fire-prone environments.

## Technical Validation

We implemented three strategies to maintain data quality. First, we conducted a detailed review of each source based on a bespoke report, showing all data and metadata, by both an AusTraits curator (primarily Wenk) and the original contributor (where possible). Measurements for each trait were plotted against all other values for the trait in AusTraits, allowing quick identification of outliers. Corrections suggested by contributors were combined back into AusTraits and made available with the next release. Version 3.0.2 of AusTraits, described here, is the sixth release.

Second, we implemented automated tests for each dataset, to confirm that values for continuous traits fall within the accepted range for the trait, and that values for categorical traits are on a list of allowed values. Data that did not pass these tests were moved to a separate spreadsheet (“excluded_data”) that is also made available for use and review.

Third, we provide a pathway for user feedback. AusTraits is an open-source community resource and we encourage engagement from users on maintaining the quality and usability of the dataset. As such, we welcome reporting of possible errors, as well as additions and edits to the online documentation for AusTraits that make using the existing data, or adding new data, easier for the community. Feedback can be posted as an issue directly at the project’s GitHub page (http://traitecoevo.github.io/austraits.build).

## Usage Notes

Each data release is available in multiple formats: first, as a compressed folder containing text files for each of the main components, second, as a compressed R object, enabling easy loading into R for those using that platform.

Using the taxon names aligned with the APC, data can be queried against location data from the Atlas of Living Australia. To create the phylogenetic tree in Fig. 6, we pruned a master tree for all higher plants^366^ using the package V.PhyloMaker^367^ and visualising via ggtree^368^. To create Fig. 3a, we used the package plotbiomes^369^ to create the baseline plot of biomes.

## Supplementary information

Supplementary Table 1

## Data Availability

All code, raw and compiled data are hosted within GitHub repositories under the Trait Ecology and Evolution organisation (http://traitecoevo.github.io/austraits.build/). The archived material includes all data sources and code for rebuilding the compiled dataset. The code used to produce this paper is available at http://github.com/traitecoevo/austraits_ms.
